# Treatment and Improved Outcomes of Three Adult Patients With Guanidinoacetate Methyltransferase (GAMT) Deficiency

**DOI:** 10.1002/jmd2.70019

**Published:** 2025-05-05

**Authors:** Angela Lee, Judith Weisenberg, Elizabeth Toolan, Marwan Shinawi

**Affiliations:** ^1^ Department of Pediatrics, Division of Genetics Children's Mercy Kansas City, UMKC School of Medicine Kansas City Missouri USA; ^2^ Departments of Pediatrics and Neurology Washington University School of Medicine Saint Louis Missouri USA; ^3^ Department of Pediatrics, Division of Genetics and Genomic Medicine Washington University School of Medicine Saint Louis Missouri USA

**Keywords:** creatine, GAMT, newborn screening, ornithine

## Abstract

Guanidinoacetate methyltransferase (GAMT) deficiency is a creatine synthesis disorder caused by biallelic pathogenic variants in *GAMT*. Early diagnosis and treatment can lead to normal neurocognitive outcomes, which has prompted its recent addition to the Recommended Uniform Screening Panel. Treatment typically includes creatine and ornithine supplementation, with or without arginine restriction or sodium benzoate. Here, we present the clinical outcomes of 3 adult patients with GAMT deficiency who began creatine and ornithine supplementation at varying ages. One patient started on treatment at 14 months of age and has had near‐normal neurocognitive outcomes, highlighting the positive clinical impact of early treatment. Our findings also emphasize the need to continue treatment throughout adulthood, but further research is required to understand the natural history and determine the optimal treatment of GAMT deficiency in adults.

**Abbreviations**
ADLs: activities of daily living
DRI: Dietary reference intake
GAA: Guanidinoacetate
GAMT: Guanidinoacetate methyltransferase
MRS: Magnetic resonance spectroscopy
RUSP: Recommended Uniform Screening Panel
SAMe: S‐adenosylmethionine

**Summary**

- Early treatment can improve neurocognitive outcome of GAMT deficiency, a treatable creatine synthesis disorder.
- Three adult patients who initiated creatine and ornithine supplementation at varying ages are reported here.
- Our data highlight the significant clinical impact of early treatment and utility of ongoing treatment in adulthood.

Guanidinoacetate methyltransferase (GAMT) deficiency was first described in 1994 [1] and has since been characterized as an autosomal recessive condition secondary to biallelic pathogenic variants in *GAMT* (OMIM *601240). The GAMT enzyme is utilized in the second step of the creatine synthesis pathway, and its absence results in the accumulation of guanidinoacetate (GAA) and creatine depletion. Clinically, this condition presents with neurodevelopmental and behavioral problems, hypotonia, seizures, as well as movement disorders.

Therapeutic response to creatine supplementation was noted in the 1990s, with normalization of creatine concentrations [1, 2]. Other interventions for GAMT‐deficiency management include ornithine or S‐adenosylmethionine supplementation, arginine restriction, and sodium benzoate therapy [3, 4, 5, 6]. Ornithine can be given at low (50–200 mg/kg/d) and high (300–800 mg/kg/d) doses. Arginine restriction varies from significant restriction of arginine to 250 mg/kg/d, giving between 0.2 and 0.5 g/kg/d of natural protein with arginine‐free amino acid formula supplementation, to protein restriction providing 0.6–1.8 g/kg of natural protein adjusted for age with or without arginine‐free amino acid formula to general reduction or avoidance of high‐protein foods [5, 7]. However, there have been no well‐designed studies to evaluate and compare the different treatment modalities. Outside of creatine and ornithine supplementation, there is no consensus regarding treatment approaches, and improvement in symptoms has been reported in various treatment regimens [5, 8, 9]. It is also unclear if adult patients with this condition should have a different course of treatment. Early diagnosis and treatment have been shown to result in normal to near normal cognitive development [5, 10, 11, 12]. The most recent addition of GAMT deficiency to the Recommended Uniform Screening Panel (RUSP) makes it even more crucial to evaluate various treatment modalities that are implemented following an early diagnosis when the patient is still asymptomatic [13, 14].

Here, we present the long‐term follow‐up of 3 adult GAMT‐deficiency patients who have largely been treated with creatine and high‐dose ornithine, including one with near‐normal neurocognitive outcomes who was started on treatment at 14 months of age. We also compare our findings to treatment modalities and outcomes of prior reported patients with GAMT deficiency who received early treatment.

## 1 Case Presentations

**FIGURE 1:**
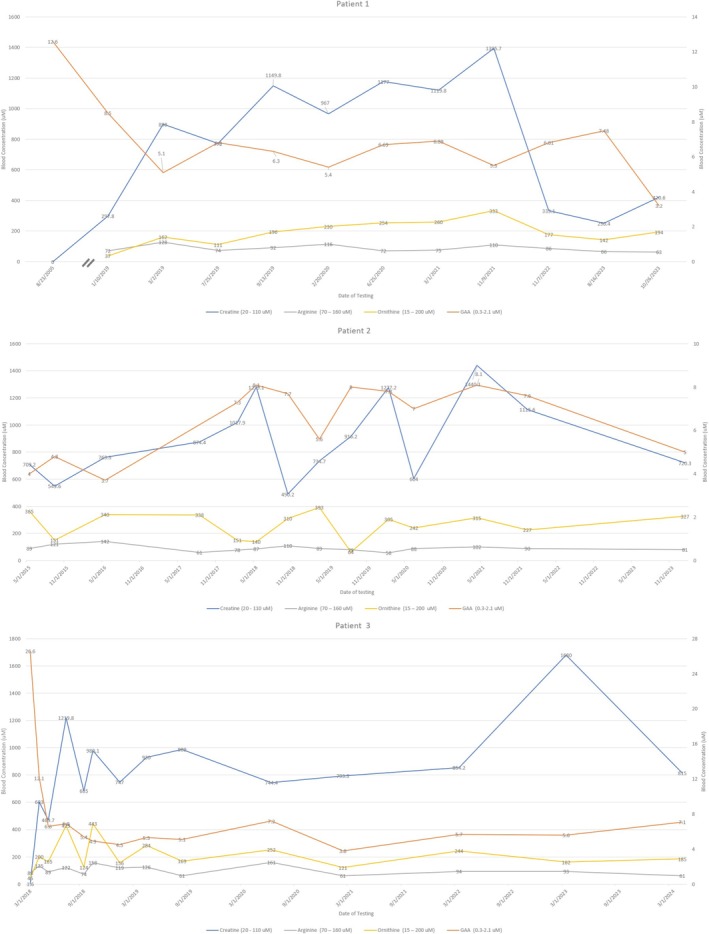
Trend of creatine, GAA, arginine, and ornithine for adult patients with GAMT in this cohort. GAA, Guanidinoacetate. μM, μmol/L.

Patient 1: Patient 1 is a 19‐year‐old male diagnosed with GAMT deficiency at 13 months of age after presentation with developmental delay, truncal instability, and hypotonia. He had elevated plasma GAA and low creatine (Figure 1 and Table 1) with diminished creatine on brain magnetic resonance spectroscopy (MRS). Genetic testing later in life revealed 2 pathogenic variants in *GAMT*: c.133T>A (p.Trp45Arg) and c.327G>A (p.Lys109=).

**TABLE 1:** Comparison of patients who were initiated on early treatment for GAMT, including patient 1 and historical patients in the literature.

Patient	Pt 1 (current cohort)	(19)	(14) pt. 1	(14) pt. 2	(5) pt. 4	(5) pt. 7	(6) pt. 1 (5) pt. 8	(6) pt. 5 (5) pt. 2	(6) pt. 4 (5) pt. 6	(11) pt. 2 (5) pt. 5 (10) pt. 4^a^	(11) pt. 3 (5) pt. 1^a^	(10) pt. 7	(12) (11) case 1 (5) pt3
Sex	M	F	—	—	F	F	F	M	M	M	F	M	F
Genotype	c.133T>A (p.Trp45Arg) AND c.327G>A (p.Lys109=)	c.442dupC (p.Gln148Profs*43) AND null allele per RNA studies	c.327G>A (p.Lys109=) AND c.522G>A (p.Trp174Ter)	Homozygous c.609dupG (p.Arg204Glufs*63)	Homozygous c.327G>A (p.Lys109=)	Unknown	c.327G>A (p.Lys109=) AND c.403G>A (p.Asp135Asn)	c.299_c.311 dup13 (p.Arg105Glyfs*26) AND c.233T>A (p.Val78Glu)	c.327G>A (p.Lys109=) AND c.522G>A (p.Trp174Ter)	c.327G>A (p.Lys109=) AND c.522G>A (p.Trp174Ter)	c.327G>A (p.Lys109=) AND c.522G>A (p.Trp174Ter)	Homozygous c.327G>A (p.Lys109=)	c.526dupG (p.Glu176Glyfs*15) AND c.152A>C (p.His51Pro)
Symptoms before therapy	Mild hypotonia, DD	DD, hypotonia	None	None	Borderline DD	Mild DD, mild epilepsy	Severe DD	None	Moderate DD, mild epilepsy	Mild DD, mild epilepsy	None	None	None
Creatine pre‐tx (μM, blood)	0 (30–120)		16.6 (37–117)	9.5 (37–117)			2 (37–117)	19 (37–117)	0.4 (37–117)	1.2 (34–130)		33.1 (34–130)	5.5^b^ (50–124)
GAA pre‐tx (μM, blood)	12.6 (0.4–3.7)		9.2 (0.5–1.8)	13.2 (0.5–1.8)			13.8 (0.5–1.8)	14.2 (0.5–1.8)	15.1 (0.5–1.8)	14.3 (0.7–5.4)	7.3 (< 2.4)	14.7 (0.7–5.4)	8.7^b^ (0.2–1.5)
Age at start of therapy	14 months	15 months	11 days	17 days	9 months	14 months	15 months	1 week	11 months	10 months	Prenatal	8 days	22 days
Therapy: Creatine mg/kg/d	400–500	265–400	500	500	300–800	300–800	300–800	300–800	300–800	300–350	350	400–600	400
Therapy: Ornithine mg/kd/d	< 100, 500–600 at 14 years	100–368	500	500	> 200–800	> 200–800	> 200–800	> 200–800	> 200–800	200–250	290	400–600	800
Therapy: Arginine restriction	No formal restriction with avoidance of high protein foods	None	Moderate protein restriction, 20%–25% kcal as protein‐free formula	Moderate protein restriction, 20%–25% kcal as protein‐free formula	Protein restriction initially with amino acid formula, later liberalized to near DRI and amino acid formula discontinued	None	Protein restriction near DRI without amino acid formula	Protein restriction near DRI without amino acid formula	Protein restriction near DRI without amino acid formula	Protein restriction near DRI without amino acid formula	Protein restriction near DRI without amino acid formula	None	0.6 g/kg/d natural protein AND 1 g/kg/d essential amino acids initially, later no formal restriction with avoidance of high protein foods
Therapy: other	SAMe from 2 to 14 years		SB 100 mg/kg/d	SB 100 mg/kg/d	SB 100 mg/kg/d	SAMe		SB 100 mg/kg/d	SB 100 mg/kg/d				± SB 100 mg/kg/d
Age at f/u	19 years	15 years 9 months	7 months	4 months	30 months	8 years 6 months	11 years 4 months	14 months	4 years 11 months	31–49 months	3 years 5 months	11 months	8 years
Creatine post‐tx (μM, blood)	798^b^ (20–110)	324.2^b^ (17–150)								1173 (34–130)		161 (34–130)	402^b^ (50–124)
GAA post‐tx (μM, blood)	6.2^b^ (0.3–2.1)	6^b^ (0.5–4.4)								3.7 (0.7–5.4)	4.5^b^ (< 2.4)	8.9 (0.7–5.4)	4^b^ (0.2–1.5)
Outcomes	Normal intellectual ability. FSIQ 96 (WAIS‐IV) Autistic features‐restrictive interests and social difficulties, ADHD, speech/articulation concerns	Mild dysdiadochokinesis and axial hypotonia. At grade level (9th grade)	Normal	Normal	DD‐speech	DD	DD‐speech and motor, mild epilepsy, autistic features	Normal	DD‐motor and speech, mild epilepsy	Improved tone, more alert, 2 word sentences. DD, mild epilepsy	Normal	DD‐no speech, hypotonia	Normal development at 14 and 31 months. Borderline DD at 8 years. IQ 71 at 8.6 years.

*Note:* (n) Reference number.

Abbreviations: μM, μmol/L; DD, developmental delay; DRI, Dietary reference intake; FSIQ, Full scale intelligence quotient; GAA, Guanidinoacetate; SAMe, S‐adenosylmethionine; SB, sodium benzoate; WAIS‐IV, Wechsler Adult Intelligence Scale IV.

^a^ Siblings.

^b^ For patients with multiple values of creatine or GAA, values were averaged pre‐/post‐treatment. In case descriptions, mild epilepsy was defined as a patient having occasional (e.g., fever induced) seizures and/or seizure freedom upon pharmacological treatment.

He sat unsupported at 10 months, used pincer grasp at 13–14 months, and had no words at 12 months. At 14 months, he was started on creatine, low‐dose ornithine, and S‐adenosylmethionine (SAMe). He discontinued ornithine for several years due to palatability concerns. At 14 years old, he transferred care to our institution, was restarted on high‐dose ornithine (400–800 mg/kg/day divided TID) to achieve GAA below 6 μM, continued receiving creatine monohydrate (400–800 mg/kg/day divided TID) to achieve normal or higher than upper normal plasma creatine levels, and SAMe was discontinued. His post‐treatment average GAA and creatine were 6.2 μM/L (reference range 0.3–2.1 μM/L) and 798 μM/L (reference range 20–110 μM/L), respectively (Figure 1).

He experienced spells concerning for partial seizures at 5.5 years old that resolved with Keppra, which was later weaned. He had one tonic–clonic seizure at 12 years old in the setting of febrile illness for which Keppra was resumed and subsequently weaned. He has remained seizure free despite being off of antiseizure medications. His adherence to GAMT‐deficiency treatment, as well as GAA and creatine levels during this time is unclear.

He is currently in college studying sports and business management and is an A‐B student. He has ADHD, anxiety, and had previously worked with speech therapy on articulation concerns. He underwent neuropsychological testing at 14 years of age that showed average academic skills and intellectual ability with borderline‐impaired visual spatial and fluid reasoning skills and concerns for features of autism with restrictive interests and social difficulties. Repeat neuropsychological testing at 19 years of age showed FSIQ of 96 (39th percentile, Wechsler Adult Intelligence Scale, 4th Edition), with verbal comprehension, nonverbal reasoning, working memory, and processing speed all in the average range. Testing was also consistent with mild ADHD and mild level 1 autism. He can perform all activities of daily living (ADLs) on his own. He is active in track, basketball, and cross country. Per prior scoring criteria [4, 9], patient 1 had a pre‐treatment severity score of 2 and a post‐treatment severity score of 0–1.

Patient 2: Patient 2 is a 21‐year‐old female who was diagnosed with GAMT deficiency at 12.5 years old. She presented with intractable epilepsy, global developmental delay, and autism. At presentation, she had no expressive language, limited eye contact, wide‐based gait, and significant behavioral concerns. She started having intermittent seizures at 3 years old that continued to worsen, despite multiple antiseizure c medications. Before diagnosis, she had multiple seizures daily associated with significant fatigue and postictal symptoms, contributing to limitations in ambulation and strength, prompting wheelchair use. Genetic testing revealed two pathogenic variants in *GAMT*: c.268G>C (p.Glu90Gln) and c.522G>A (p.Trp174*) variants.

She started on creatine (400–800 mg/kg/day) and high‐dose ornithine (400–800 mg/kg/day to achieve GAA level < 6 μM) at 12 years old. A protein restriction was attempted; however, the patient enjoyed protein rich foods and had an intake around 1.5–2.5 g/kg/d. She was unable to tolerate a trial with sodium benzoate for 1 week due to side effects. Within 6 months of treatment, seizures resolved and all antiseizur medications were weaned. She had improved muscle tone and acquired stable gait and ability to climb stairs independently, self‐feed, and partially toilet train. She continues to be mostly nonverbal; however, she is more socially communicative and shows more interest in activities and school. She continues to need assistance with ADLs. Her post‐treatment average GAA and creatine were 6.6 μM/L (reference range 0.3–2.1 μM/L) and 935 μM/L (reference range 20–110 μM/L), respectively (Figure 1). Her pre‐treatment severity score was 6, decreasing to 3 post‐treatment.

Patient 3: Patient 3 is a 24‐year‐old female who was diagnosed with GAMT deficiency at 19 years old. She presented with severe intellectual disability, autism, and epilepsy. She was non‐verbal, unable to perform ADLs, and had epilepsy since 6 years of age that required multiple antiseizure medications. Genetic testing revealed the following pathogenic variants in *GAMT*: c.299_311dup13 (p.Arg105Glyfs*26) and c.327G>A (p.Lys109=). She was started on creatine and high‐dose ornithine at diagnosis. She began limiting consumption of high‐protein foods while meeting the dietary reference intake (DRI) for age. With treatment, she showed significant improvement in her interaction, has learned several signs, and seizures resolved, prompting weaning of all antiseizure medications. Her pre‐treatment plasma GAA and creatine levels went from 26.6 to 1.6 μM/L, respectively, to average post‐treatment levels of 6.2 and 885 μM/L, respectively (Figure 1). Her pre‐treatment severity score was 6, decreasing to 3 post‐treatment.

## 2 Discussion

All patients showed significant clinical improvement with creatine and high‐dose ornithine. In addition, all patients were weaned off antiseizure medications when their GAA values were reduced and maintained below 6–7 μM. Although there have been no reported target creatine levels, we aimed to maintain a plasma level > 800 μM. Sodium benzoate was discussed with all patients and attempted in one patient, but it was not tolerated. Arginine or protein restriction was also discussed with families, with one patient limiting high‐protein foods; however, no patients were on significant arginine restriction or supplemental amino acid formula. In the literature, there is a spectrum of treatment modalities instituted, age of treatment onset, and overall outcomes. Most patients are initiated on creatine and high‐dose ornithine, but the addition of sodium benzoate and the level of arginine restriction varies significantly. While arginine restriction has a biologic basis, clinically there is a mixed response to arginine restriction [2, 5, 15]. The same is true for sodium benzoate [6, 16]. Both of these treatment options can come with significant challenges. Sodium benzoate is unpalatable and is associated with unpleasant GI side effects. Significant arginine or protein restriction is a major dietary modification that has adherence challenges, while protein restriction to near the DRI or limitation of high‐protein foods can be more manageable. Additional studies on the benefits of each treatment modality are needed; however, the feasibility of this can be limited.

Previous prevalence estimates for GAMT deficiency range from 1 in 114 072 to 1 in 2 640 000 [6, 17]. GeniE, the Genetic Prevalence Estimator, supported by the Broad Institute, estimates a global prevalence of 1 in 845 097, using data from ClinVar (March 7, 2024 release) and GnomAD (version 4.0.0) [18]. Although these estimates vary widely and are impacted by various factors, it does seem to suggest an increased prevalence compared with currently published GAMT‐deficiency cases. Notably, two of our patients were not diagnosed until much later in life after a long diagnostic journey, highlighting the need for clinical suspicion for this rare treatment‐responsive metabolic disorder and the potential benefit of newborn screening.

In comparison with previously reported GAMT‐deficiency cases with relatively early diagnosis and treatment (< 15 months of age) (Table 1), patient 1 has had the longest reported follow‐up, although many of the prior reported patients are likely now near adulthood. He has overall done very well, although he has some features of autism and behavioral concerns that may be related to several factors. Initiation of treatment was delayed by diagnosis at 13–14 months of age. He was on low‐dose ornithine that was then discontinued for years, before restarting high‐dose ornithine at 14 years old. Additionally, he was not treated with significant arginine restriction or glycine reducing therapy. Interestingly, he developed epilepsy while on creatine and SAMe, although epilepsy often responds well to creatine supplementation in this condition. He was not on ornithine, arginine restriction, or glycine reducing therapy at the time. These therapies are thought to contribute to a decrease in neurotoxic GAA, potentially playing a role in epilepsy. In addition, his adherence during this time is unclear and GAA and creatine levels were unavailable at these times. With current treatment, he has not had recurrence of seizures off of other antiseizure medications, suggesting that these aspects may have had an impact on previous episodes of epilepsy while on creatine and SAMe. Previous cohorts have noted developmental delay in those treated after 9 months of age despite various interventions, as well as improved outcomes in those treated before 1 month of age (Table 1) suggesting that treatment should be initiated as early as possible [5, 6, 10, 11, 12]. With anticipated addition to newborn screening in states across the country, there can be a range of therapies instituted after diagnosis. A recently reported GAMT‐deficiency patient, with more extensive follow‐up after early treatment, showed minimal symptoms after over 15 years of treatment with high‐dose ornithine and creatine alone [19]. Our patient cohort, particularly patient 1, as well as cases in the literature [5, 11, 19] provide long‐term clinical data on the significant impact of creatine and high‐dose ornithine alone, although it may be prudent to be cautious with significant arginine restriction and sodium benzoate in patients initially identified on newborn screen. Our results also highlight the need for continuing therapy throughout adulthood to control seizures and maintain other positive outcomes including improved muscle tone and interaction, as well as the ability to perform certain daily life activities. This is also noted in the literature [5, 20, 21]; however, treatment modalities differ more significantly in previously reported adults, including some on no treatment or creatine alone [5, 20]. Over time, research into the necessity of various treatment modalities for normal neurocognitive outcomes will be needed.

## Author Contributions

Dr. Lee gathered clinical information and prepared the manuscript. Drs. Weisenberg, Shinawi, and Liz Toolan gathered clinical information and revised the manuscript.

## Ethics Statement

The authors have nothing to report.

## Consent

Consent for publication was obtained in writing from all patients in this case series or their guardians.

## Conflicts of Interest

The authors declare no conflicts of interest.

## Data Availability

Additional data is available upon reasonable request of the corresponding author.

## References

[1] S. Stockler , U. Holzbach , F. Hanefeld , et al., “Creatine Deficiency in the Brain: A New, Treatable Inborn Error of Metabolism,” Pediatric Research 36, no. 3 (1994): 409–413. 7808840 10.1203/00006450-199409000-00023

[2] S. Stockler , F. Hanefeld , and J. Frahm , “Creatine Replacement Therapy in Guanidinoacetate Methyltransferase Deficiency, a Novel Inborn Error of Metabolism,” Lancet 348, no. 9030 (1996): 789–790. 8813986 10.1016/s0140-6736(96)04116-5

[3] R. Ensenauer , T. Thiel , K. O. Schwab , et al., “Guanidinoacetate Methyltransferase Deficiency: Differences of Creatine Uptake in Human Brain and Muscle,” Molecular Genetics and Metabolism 82, no. 3 (2004): 208–213. 15234333 10.1016/j.ymgme.2004.04.005

[4] S. Mercimek‐Mahmutoglu , S. Stoeckler‐Ipsiroglu , A. Adami , et al., “GAMT Deficiency: Features, Treatment, and Outcome in an Inborn Error of Creatine Synthesis,” Neurology 67, no. 3 (2006): 480–484, 10.1212/01.wnl.0000234852.43688.bf. 16855203

[5] S. Stockler‐Ipsiroglu , C. van Karnebeek , N. Longo , et al., “Guanidinoacetate Methyltransferase (GAMT) Deficiency: Outcomes in 48 Individuals and Recommendations for Diagnosis, Treatment and Monitoring,” Molecular Genetics and Metabolism 111, no. 1 (2014): 16–25, 10.1016/j.ymgme.2013.10.018. 24268530

[6] K. S. Viau , S. L. Ernst , M. Pasquali , L. D. Botto , G. Hedlund , and N. Longo , “Evidence‐Based Treatment of Guanidinoacetate Methyltransferase (GAMT) Deficiency,” Molecular Genetics and Metabolism 110, no. 3 (2013): 255–262. 24071436 10.1016/j.ymgme.2013.08.020

[7] G. Fernandes‐Pires and O. Braissant , “Current and Potential New Treatment Strategies for Creatine Deficiency Syndromes,” Molecular Genetics and Metabolism 135, no. 1 (2022): 15–26. 34972654 10.1016/j.ymgme.2021.12.005

[8] O. Braissant , “GAMT Deficiency: 20 Years of a Treatable Inborn Error of Metabolism,” Molecular Genetics and Metabolism 111, no. 1 (2014): 1–3. 24275206 10.1016/j.ymgme.2013.11.002

[9] Y. Khaikin , S. Sidky , J. Abdenur , et al., “Treatment Outcome of Twenty‐Two Patients With Guanidinoacetate Methyltransferase Deficiency: An International Retrospective Cohort Study,” European Journal of Paediatric Neurology 22, no. 3 (2018): 369–379. 29506905 10.1016/j.ejpn.2018.02.007

[10] S. U. Dhar , F. Scaglia , F. Y. Li , et al., “Expanded Clinical and Molecular Spectrum of Guanidinoacetate Methyltransferase (GAMT) Deficiency,” Molecular Genetics and Metabolism 96, no. 1 (2009): 38–43. 19027335 10.1016/j.ymgme.2008.10.008

[11] A. H. El‐Gharbawy , J. L. Goldstein , D. S. Millington , et al., “Elevation of Guanidinoacetate in Newborn Dried Blood Spots and Impact of Early Treatment in GAMT Deficiency,” Molecular Genetics and Metabolism 109, no. 2 (2013): 215–217. 23583224 10.1016/j.ymgme.2013.03.003

[12] A. Schulze , G. F. Hoffmann , P. Bachert , et al., “Presymptomatic Treatment of Neonatal Guanidinoacetate Methyltransferase Deficiency,” Neurology 67, no. 4 (2006): 719–721. 16924036 10.1212/01.wnl.0000230152.25203.01

[13] “Newborn Screening for Guanidinoacetate Methyltransferase (GAMT) Deficiency: A Summary of the Evidence and Advisory Committee Decision 2022,” accessed April 30, 2023, https://www.hrsa.gov/sites/default/files/hrsa/advisory‐committees/heritable‐disorders/rusp/gamt‐deficiency‐consumer‐summary.pdf.

[14] K. Hart , A. Rohrwasser , H. Wallis , et al., “Prospective Identification by Neonatal Screening of Patients With Guanidinoacetate Methyltransferase Deficiency,” Molecular Genetics and Metabolism 134, no. 1–2 (2021): 60–64. 34389248 10.1016/j.ymgme.2021.07.012

[15] S. Mercimek‐Mahmutoglu , M. Dunbar , A. Friesen , et al., “Evaluation of Two Year Treatment Outcome and Limited Impact of Arginine Restriction in a Patient With GAMT Deficiency,” Molecular Genetics and Metabolism 105, no. 1 (2012): 155–158. 22019491 10.1016/j.ymgme.2011.09.037

[16] S. Mercimek‐Mahmutoglu , G. S. Salomons , and A. Chan , “Case Study for the Evaluation of Current Treatment Recommendations of Guanidinoacetate Methyltransferase Deficiency: Ineffectiveness of Sodium Benzoate,” Pediatric Neurology 51, no. 1 (2014): 133–137. 24766785 10.1016/j.pediatrneurol.2014.02.011

[17] C. L. Desroches , J. Patel , P. Wang , et al., “Carrier Frequency of Guanidinoacetate Methyltransferase Deficiency in the General Population by Functional Characterization of Missense Variants in the GAMT Gene,” Molecular Genetics and Genomics 290, no. 6 (2015): 2163–2171. 26003046 10.1007/s00438-015-1067-x

[18] Broad Institute , “GAMT—Dashboard,” https://genie.broadinstitute.org/dashboard/ENSG00000130005, (2024).

[19] L. M. Marten , R. Kratzner , G. S. Salomons , et al., “Long Term Follow‐Up in GAMT Deficiency—Correlation of Therapy Regimen, Biochemical and In Vivo Brain Proton MR Spectroscopy Data,” Molecular Genetics and Metabolism Reports 38 (2024): 101053. 38469086 10.1016/j.ymgmr.2024.101053 PMC10926185

[20] B. P. Modi , H. N. Khan , R. van der Lee , et al., “Adult GAMT Deficiency: A Literature Review and Report of Two Siblings,” Molecular Genetics and Metabolism Reports 27 (2021): 100761. 33996490 10.1016/j.ymgmr.2021.100761 PMC8093930

[21] A. Schulze , P. Bachert , H. Schlemmer , et al., “Lack of Creatine in Muscle and Brain in an Adult With GAMT Deficiency,” Annals of Neurology 53, no. 2 (2003): 248–251. 12557293 10.1002/ana.10455

